# Emergency Management of Anaphylaxis: A High Fidelity Interprofessional Simulation Scenario to Foster Teamwork Among Senior Nursing, Medicine, and Pharmacy Undergraduate Students

**DOI:** 10.7759/cureus.2915

**Published:** 2018-07-03

**Authors:** Sandra MacDonald, April Manuel, Adam Dubrowski, Natalie Bandrauk, Rebecca Law, Vernon Curran, Young Wah Lee

**Affiliations:** 1 School of Nursing, Memorial University of Newfoundland, St. John's, CAN; 2 School of Nursing, Memorial University of Newfoundland, Conception Bay South, CAN; 3 Emergency Medicine, Memorial University of Newfoundland, St. John's, CAN; 4 Faculty of Medicine, Memorial University of Newfoundland, St. John's, CAN; 5 School of Pharmacy, Memorial University of Newfoundland, St. John's, CAN; 6 Faculty of Medicine, Memorial University of Newfoundland

**Keywords:** high-fidelity simulation, interprofessional education, anaphylaxis, nursing, pharmacy, medicine, undergraduate education

## Abstract

Nursing, medicine, and pharmacy students have limited opportunities during their undergraduate programs to learn and practice together as an interprofessional team. This has prompted faculty at Memorial University of Newfoundland to explore the use of high fidelity simulated interprofessional education (HF-IPE) to help nursing, medicine, and pharmacy students learn about their roles, develop communication and collaboration skills, and foster teamwork. Research has shown that high fidelity simulated education can promote critical thinking, engage learners, improve confidence, and enhance psychomotor skills; however, there is limited data on the impact of HF-IPE on fostering teamwork. This technical report describes one HF-IPE scenario designed to foster teamwork among senior undergraduate nursing, medicine, and pharmacy students. The scenario is designed to promote an understanding of the roles of nursing, medical, and pharmaceutical professionals in an interprofessional team during the emergency management of an adult patient experiencing acute anaphylaxis. Teamwork and communication skills are emphasized, and students are provided with the opportunity to communicate and collaborate within an interprofessional healthcare team.

## Introduction

Teamwork which involves learning with, from, and about one another is an important and requisite skill that must be taught, supported, and nurtured in undergraduate health sciences programs [1-4]. Currently, nursing, medicine, and pharmacy students have few opportunities to practice interprofessional teamwork during their undergraduate education, and they often enter the workforce with little or no experience in working with interprofessional healthcare teams [5-6]. This has been further complicated by a lack of opportunities to practice teamwork behaviors in the clinical setting. These concerns have prompted educators and researchers to search for appropriate and effective teaching and learning strategies to prepare senior students for real-life situations as members of the healthcare team [7]. 

One effective teaching and learning strategy known to foster teamwork among undergraduate health sciences students is simulated interprofessional education [8-9]. In particular, high fidelity simulated interprofessional education (HF-IPE) has been shown to effectively promote skill acquisition, enhance clinical judgment, and teach students about complex clinical situations [10-14]. HF-IPE can provide an invaluable safety net for learning, allowing students to acquire and develop critical-thinking and decision-making skills without exposing patients or students to unnecessary risk [15-18].

The HF-IPE scenario discussed in this report is designed to create a safe learning environment for undergraduate students to learn with and from one another while managing the care of an adult patient experiencing acute anaphylaxis. This HF-IPE scenario was developed using the Jeffries simulation framework for designing, implementing, and evaluating simulation education. The Jeffries framework includes five major components: the simulation design, learner outcomes, faculty role, student characteristics, and educational practices [19]. The simulation design for this scenario is high fidelity, with learner outcomes including enhanced knowledge of the roles of nursing, medical, and pharmaceutical professionals on the team, improved attitudes towards teamwork, and the demonstration of teamwork behaviors while caring for a patient experiencing anaphylaxis. The learning experience is student-centered, while the faculty play the role of facilitator and observer. The educational practices include building on students' previous knowledge and skills related to teamwork to create new knowledge, behaviors, and skills.

The World Allergy Organization Guidelines for the assessment and management of anaphylaxis are used to guide the simulation, thus creating an awareness of best practice protocols for managing acute anaphylaxis, as well as an opportunity to foster teamwork among undergraduate nursing, medicine, and pharmacy students [20].

## Technical report

**Figure 1:**
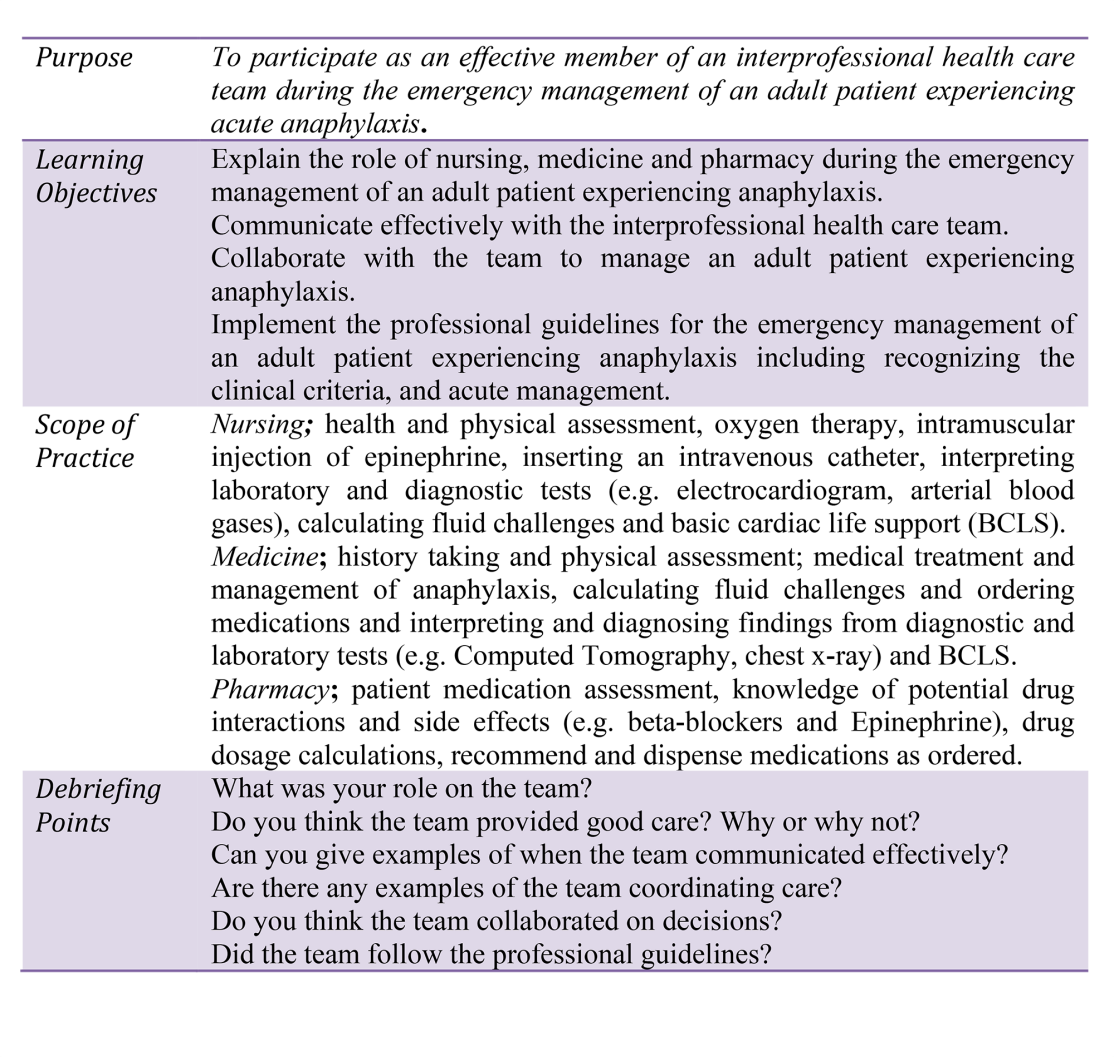
Anaphylaxis Teaching Plan

There are three healthcare professional education faculties and schools at Memorial University of Newfoundland: nursing, medicine, and pharmacy, all of which are dedicated to the development of undergraduate and postgraduate students. However, currently, there are few opportunities for these groups of undergraduate students to interact face to face as required during common interprofessional activities. The purpose of implementing the HF-IPE scenario described in this technical report is to provide undergraduate nursing, medical, and pharmacy students with an opportunity to build on previous knowledge and skills by participating as effective members of an interprofessional healthcare team during the management of an adult patient experiencing acute anaphylaxis. This scenario is designed to be implemented within any senior level clinical course. The purpose, learning objectives, scope of practice and debriefing points for the scenario are outlined in Figure 1.

Inputs

1.      Senior undergraduate nursing, medicine, and pharmacy students

2.     Nursing, medicine, and pharmacy interprofessional educators

3.     Adult female/male high fidelity human patient simulator (HPS) programmed to reflect the five stages of the simulation: (1) baseline assessment, (2) early reaction, (3) anaphylaxis, (4) recovery and (5) resolution

4.     Mock chart: a paper-based chart containing the patient's history and physical exam notes, progress notes, nursing kardex, medication orders, physician orders, vital signs record, medication administration record, laboratory and diagnostics tests, and anaphylaxis protocol

5.     Equipment: cardiac monitor, pulse oximeter, Venturi mask, intravenous (IV) access, nebulizer and face mask, intravenous fluids, IV set-up tray, mannequin make up for urticaria on abdomen

Processes

**Figure 2:**
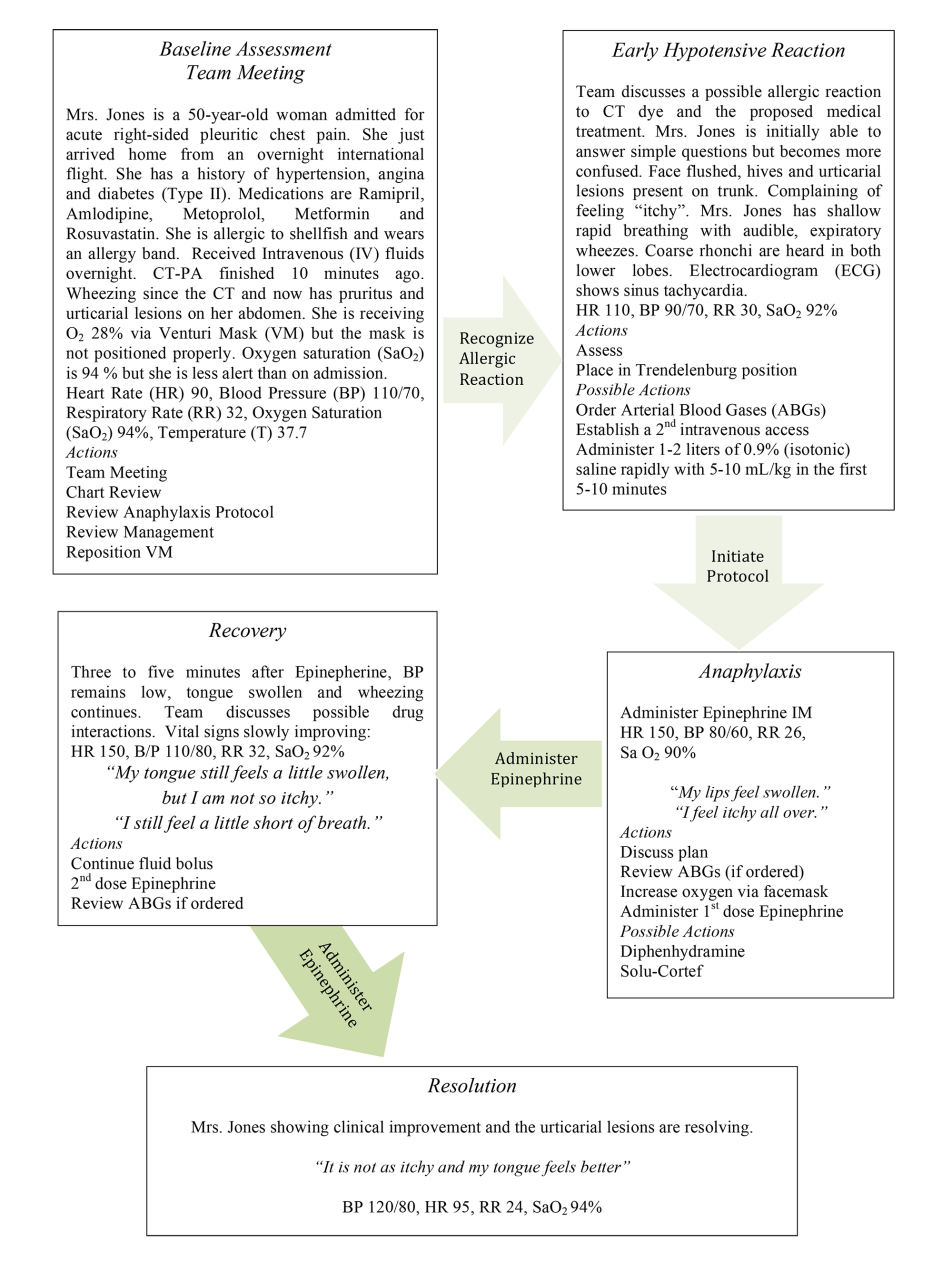
Story Board for Acute Anaphylaxis VS - vital signs; HR - heart rate; BP - blood pressure; RR - respiratory rate; T - temperature; CT - computed tomography; ECG - electrocardiogram; SaO2 - oxygen saturation; ABG - arterial blood gas; IM - intramuscular; VM - Venturi mask, and IV - intravenous.

**Figure 3:**
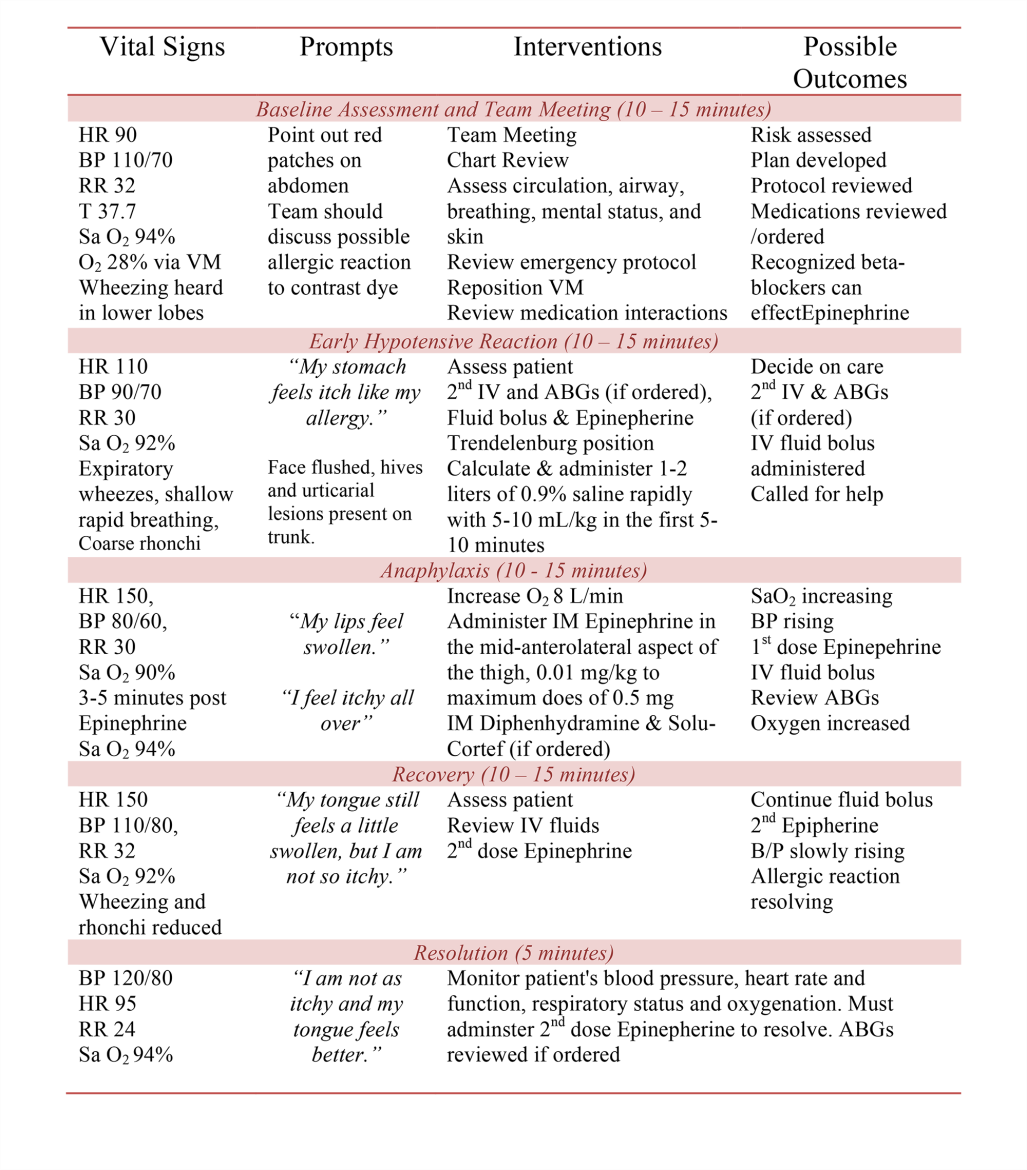
Story Board for Programming Human Patient Simulator VS - vital signs; HR - heart rate; BP - blood pressure; RR - respiratory rate; T - temperature; CT - computed tomography; ECG - electrocardiogram; SaO2 - oxygen saturation; ABG - arterial blood gas; IM - intramuscular; VM - Venturi mask, and IV - intravenous.

The story board developed to guide the implementation of the simulation is divided into five phases: (1) baseline assessment, (2) early hypotensive reaction, (3) anaphylaxis, (4) recovery, and (5) resolution (Figure 2). Each phase of the simulation includes a description of the situation, assessment data, prompts, and possible actions. Further information for programming the human patient simulator is also provided in the 'Story Board for Programming the Human Patient Simulator' including vital signs and cardiac and respiratory status (Figure 3).

## Discussion

Teamwork is an important and requisite skill that must be taught, supported, and nurtured in nursing, medicine, and pharmacy undergraduate education programs. Although there are three health professional education faculties and schools at Memorial University of Newfoundland, nursing, medicine, and pharmacy, currently there are few opportunities for these groups of undergraduate students to interact face-to-face during common interprofessional activities. This is not unique to Memorial, and similar early introductions to teamwork and communication at the undergraduate learners’ level are absent in other universities. This has led faculty at Memorial to develop a HF-IPE scenario to foster teamwork; however, barriers to implementing this scenario exist, including common challenges associated with implementing IPE in the academic setting: difficulties in scheduling and in matching students’ scope of practice.

The professional nursing, medicine, and pharmacy courses have rigid and busy schedules with little or no time for interprofessional activities, unless they can be scheduled within a course. In addition, the scope of practice for each of the professions varies depending on the level of the student, e.g., junior or senior. This HF-IPE scenario is designed for senior-level students, but when there are different levels of students participating, certain modifications may be needed. For example, junior medical students may be aware of the practice protocols for anaphylaxis, but they may have little or no experience managing the treatment of acute anaphylaxis; in contrast, senior medical students would be more likely to have both, knowledge of protocols as well as experience in managing a deteriorating patient in the clinical setting. This varying scope of practice could affect the functioning of the team and have an impact on their learning about their roles.

In order to offset some of these limitations, this simulation scenario is developed for senior-level students and focuses on fostering teamwork in addition to developing proficiency in managing anaphylaxis in a deteriorating patient. Prior to participating in the simulation, students are given time to review the anaphylaxis protocols and mock chart and ask questions about their role in the team. The focus of this simulation is teamwork, which is also emphasized in the discussion questions that guide the debriefing session: e.g., do you think the team communicated effectively? In this way, this scenario is designed to help educators foster teamwork among senior students and promote an understanding of their role in the interprofessional team during the management of an adult patient experiencing acute anaphylaxis. Teamwork and communication skills are emphasized in this scenario, by providing students with an opportunity to communicate and collaborate face-to-face with an interprofessional healthcare team. Best practice guidelines for the emergency management of an adult patient experiencing acute anaphylaxis are used to guide the simulation, thus creating an awareness of best practice protocols for managing acute anaphylaxis, as well as creating an opportunity for undergraduate nursing, medicine, and pharmacy undergraduate students to learn with, from, and about one other.

## Conclusions

The purpose of this technical report is to provide educators who use HF-IPE with a detailed teaching plan and story boards for implementing an HF-IPE scenario to foster teamwork among senior nursing, medicine, and pharmacy undergraduate students. This report is designed to provide the reader with the context, required resources, scenario, learning objectives, and possible outcomes for a high fidelity simulation designed to care for an adult patient experiencing acute anaphylaxis. Teamwork and communication skills are emphasized while students are provided with the opportunity to learn with, from, and about the nursing, medical, and pharmaceutical members of the interprofessional healthcare team. Best practice guidelines for the emergency management of an adult patient experiencing acute anaphylaxis are used to guide teamwork behaviors during the simulation.
